# The In Vivo Antioxidant and Hepatoprotective Actions of Selected Chinese Teas

**DOI:** 10.3390/foods9030262

**Published:** 2020-03-02

**Authors:** Shi-Yu Cao, Bang-Yan Li, Ren-You Gan, Qian-Qian Mao, Yuan-Feng Wang, Ao Shang, Jin-Ming Meng, Xiao-Yu Xu, Xin-Lin Wei, Hua-Bin Li

**Affiliations:** 1 Guangdong Provincial Key Laboratory of Food, Nutrition and Health, Department of Nutrition, School of Public Health, Sun Yat-Sen University, Guangzhou 510080, China; caoshy3@mail2.sysu.edu.cn (S.-Y.C.); liby35@mail2.sysu.edu.cn (B.-Y.L.); maoqq@mail2.sysu.edu.cn (Q.-Q.M.); shangao@mail2.sysu.edu.cn (A.S.); mengjm@mail2.sysu.edu.cn (J.-M.M.); xuxy53@mail2.sysu.edu.cn (X.-Y.X.); 2 Research Center for Plants and Human Health, Institute of Urban Agriculture, Chinese Academy of Agricultural Sciences, Chengdu 610213, China; ganrenyou@caas.cn; 3 College of Life Sciences, Shanghai Normal University, 100 Guilin Road, Shanghai 200234, China; yfwang@shnu.edu.cn; 4 Department of Food Science & Technology, School of Agriculture and Biology, Shanghai Jiao Tong University, Shanghai 200240, China; weixinlin@sjtu.edu.cn

**Keywords:** *Camellia sinensis*, tea, antioxidant activity, hepatoprotective effect, alcohol, catechins, polyphenol, chlorogenic acid

## Abstract

Tea is a popular beverage and shows very strong in vitro antioxidant activity. However, the relationship among in vitro and in vivo antioxidant activities in teas is seldom reported. In this study, in vivo antioxidant and hepatoprotective activities of 32 selected Chinese teas were evaluated on a mouse model with acute alcohol-induced liver injury. The results showed that most teas significantly reduced the levels of alanine transaminase, aspartate transaminase, alkaline phosphatase, triacylglycerol, and total bilirubin in the sera of mice at a dose of 400 mg/kg. In addition, most teas greatly decreased the malondialdehyde level and increased the levels of superoxide dismutase, glutathione peroxidase, and glutathione in the liver of mice, indicating the antioxidant and hepatoprotective activities of teas. Furthermore, the in vivo antioxidant activity of dark tea was stronger than that of green tea, opposite to the results of the in vitro study. Among these 32 teas, Black Fu Brick Tea, Pu-erh Tea, and Qing Brick Tea showed the strongest antioxidant and hepatoprotective activities. Moreover, total phenolic content as well as the contents of epicatechin, gallocatechin gallate, and chlorogenic acid were found to contribute, at least partially, to the antioxidant and hepatoprotective actions of these teas. Overall, teas are good dietary components with antioxidant and hepatoprotective actions.

## 1. Introduction

Reactive oxygen species (ROS) are necessary for normal metabolism and act as specific signaling factors under physiological conditions [1]. However, excessive free radicals can damage cell lipids, proteins, and DNA, leading to certain chronic diseases such as cardiovascular diseases, cancers, and liver diseases [2].

Excessive alcohol consumption is a global health concern. According to a World Health Organization (WHO) [3] report, excessive alcohol consumption is responsible for over 3 million deaths globally each year. Excessive alcohol consumption can lead to many medical issues such as nausea, gastrointestinal diseases, alcoholic liver disease, diabetes mellitus, and cancer [2,4]. Alcoholic liver disease is attracting increasing attention due to the fact of its high morbidity and mortality. Alcohol consumption can induce the generation of ROS such as superoxide anion radical, hydroxyl radical, and hydrogen peroxide [5]. Therefore, oxidative stress is a main factor mediated in the development of alcoholic liver disease. The antioxidant system includes enzymatic and non-enzymatic antioxidants. Antioxidant enzymes mainly comprise catalase, superoxide dismutase (SOD), and glutathione peroxidase (GPx), while glutathione (GSH), vitamin E, ascorbate, vitamin A, and ubiquinone are the main non-enzymatic antioxidants in mammals [6]. The antioxidant system of the human body can be enhanced by dietary antioxidant intake. Recently, it was reported that the use of plant extracts with antioxidant activity, instead of a single known antioxidant, can protect against oxidative stress-related diseases, such as cancer, metabolic disorder, and alcoholic liver disease, through influencing the cellular oxidative balance [4,7,8,9,10,11].

Tea is a popular drink around the world. Tea can be classified into six categories including green, white, yellow, oolong, black, and dark teas according to the degree of fermentation. Tea has many health benefits such as cardiovascular protective, anticancer, and anti-diabetic activities [12,13,14]. Tea contains many natural antioxidants such as catechins and caffeine [15]. Several studies indicate that tea possesses very strong in vitro antioxidant activity [15,16]. Although there have been several studies reporting the in vivo antioxidant effects of tea against alcoholic liver injuries, most of them focus on a single tea category, such as green tea or black tea, or a single component such as epigallocatechin gallate (EGCG) [17,18,19,20]. Besides, the in vitro antioxidant activity of natural products may be very different from the in vivo antioxidant activity, due to the differences of the bioavailability of bioactive compounds [21,22]. Therefore, it is very important to systematically evaluate the in vivo antioxidant activity of six categories of tea.

In this study, the in vivo antioxidant and hepatoprotective activities of the 32 mostly consumed Chinese teas from six categories were evaluated and compared using a mouse model with the acute alcohol-induced liver injury. In addition, several phytochemicals contributing to these bioactivities were identified. The results can instruct the public to select the tea possessing strong in vivo antioxidant activity for the prevention of oxidative stress-related chronic diseases and are also useful to develop related functional foods and pharmaceuticals to prevent and treat alcoholic liver injury as well as other oxidative stress-related diseases.

## 2. Materials and Methods

### 2.1. Chemicals and Reagents

The ethanol, acetic acid, chloral hydrate, and sodium chloride were of analytical grade and were obtained from Tianjin Chemical Factory (Tianjin, China). The methanol and formic acid were of HPLC grade and produced by Macklin Chemical Factory (Shanghai, China). Detection kits of SOD, malondialdehyde (MDA), GSH, GPx, and total protein were purchased from Nanjing Jiancheng Bioengineering Institute (Nanjing, China). The 2,4,6-tri(2-pyridyl)-s-triazine (TPTZ), 6-hydroxy-2,5,7,8-tetramethyl-chromane-2-carboxylic acid (Trolox), 2,2′-azino-bis(3-ethylbenothiazoline-6-sulphonic acid) diammonium salt (ABTS), and Folin–Ciocalteu’s phenol were obtained from Sigma–Aldrich (Saint Louis, MO, USA). The standard chemicals for HPLC analysis were produced by Derick Biotechnology Co., Ltd. (Chengdu, China), including gallocatechin (GC), epigallocatechin (EGC), catechins (C), EGCG, epicatechin (EC), gallocatechin gallate (GCG), epicatechin gallate (ECG), and catechin gallate (CG), gallic acid, chlorogenic acid, caffeine, ellagic acid, myricetin, quercitrin, astragalin, quercetin, theaflavin, and kaempferol. All other chemicals or reagents were of analytical grade, and deionized water was used for all experiments.

### 2.2. Preparation of Tea Extracts

The detailed information of 32 Chinese teas are provided in Table 1. The sample (10 g) was extracted by boiling distilled water (100 mL) in a 98 °C water bath shaker (DKZ-450B, Senxin, China) for 10 min, and the extraction was repeated three times. After extraction, all the infusions were combined and mixed. The infusions were concentrated in a vacuum rotary evaporator at 60 °C and were subsequently freeze-dried into powders using a freeze drier (Labconco-7752001, Kansas City, MO, USA). The dried extract was stored at −20 °C for further use. The extract was resuspended to 40 g/L (*w*/*v*) with distilled water before use.

### 2.3. Animal Study and Sample Preparation

The male Kunming mice (20 g) were purchased from the Laboratory Animal Center of Sun Yat-Sen University (Guangzhou, China). The mice were kept in specific pathogen-free (SPF) animal room with the temperature at 22 ± 0.5 °C, relatively humidity at 40–60%, and a 12 h light/dark cycle. The animal study was carried out according to the “Principles of Laboratory Animal Care and Use” approved by the School of Public Health, Sun Yat-Sen University (No. 2019-002; 28 February 2019).

The mice were randomly divided into different groups, including the control group, the model group, and 32 treatment groups with six mice in each group. After one week of adaptation, the mice were used for the intervention. Mice in the 32 treatment groups were administrated with 10 mL/kg body weight of tea extracts, equal to a dose of 400 mg/kg, for 15 days. This dose of tea extract was chosen according to the literature which reported that tea extracts showed good antioxidant and anti-fibrotic activities at the dose range of 100–400 mg/kg body weight [23,24,25]. The control and model groups were daily treated with distilled water (10 mL/kg) at the same time for 15 days. Thirty minutes after the last time of administration, the model group and 32 treatment groups were treated with ethanol solution (50%, *v*/*v*) twice at a dose of 10 mL/kg body weight with a 7 h interval, while the control group was treated with distilled water. All the interventions were performed by gavage. Two hours after the last time of ethanol treatment, all the mice were weighed, anesthetized by intraperitoneally injecting 10% chloral hydrate (350 mg/kg body weight), and then sacrificed.

The blood samples were collected by removing eyeballs from the mice and kept at room temperature for one hour. After that, the samples were centrifuged (3000× *g*, 4 °C, and 10 min) to collect the serum, which was then stored at 4 °C and detected by a chemistry analyzer (Hitachi-7180, Tokyo, Japan). The mouse liver tissue was also collected and weighed, and a part of it was fixed by 4% (*w*/*v*) paraformaldehyde, with the rest stored at −80 °C before use.

### 2.4. Measurement of Hepatic Injury Biochemical Markers in the Serum

The levels of alanine transaminase (ALT), aspartate transaminase (AST), alkaline phosphatase (ALP), triacylglycerol (TG), total bilirubin (TBIL), and total protein (TP) were measured by a Hitachi-7180 automated biochemistry analyzer with a diagnostic reagent kit according to the literature [22,26].

### 2.5. Measurement of Antioxidant Biochemical Markers in the Liver

Liver tissue was homogenized using an ice-cold 0.9% NaCl solution into 10% (*w*/*v*) homogenate. The supernatant was obtained by centrifugation at 3000× *g* for 10 min and used for the subsequent analysis. The levels of SOD, GPx, and GSH in the liver tissue were measured by commercial detection kits (Jiancheng, Nanjing, China) according to the previous report [22] and presented as U/ mg prot, activity unit (AU), and mg/g prot, respectively.

### 2.6. Measurement of Lipid Peroxidation Levels in the Liver

The degrees of lipid peroxidation in the liver tissue were determined by commercial detection kits (Jiancheng, Nanjing, China) based on the thiobarbituric acid (TBA) method according to the previous report [22]. The results are presented as nmol MDA equivalent/mg prot with MDA as the reference standard.

### 2.7. Histopathologic Assessment of Liver

The liver tissue was fixed in 4% paraformaldehyde, embedded in paraffin, and sliced into 5 µm thick sections before staining with hematoxylin–eosin. The histopathological assessment was performed as described in a previous study [22].

### 2.8. Measurement of Antioxidant Capacity and Total Phenolic Content

The ferric-reducing antioxidant power (FRAP) assay was applied to measure the reducing ability of tea based on the method reported by Benzie et al. [27]. Briefly, sodium acetate-acetic acid buffer (300 mmol/L), TPTZ solution (10 mmol/L), and ferric chloride solution (20 mmol/L) were mixed into the FRAP reagent with a volume ratio of 10:1:1. The 100 µL sample was added into 3 mL FRAP reagent and reacted for 4 min at room temperature before the absorbance was detected at 593 nm. The FeSO_4_ was used as a standard, and the results were recorded as µmol Fe^2+^/g dry weight (DW).

The Trolox equivalent antioxidant capacity (TEAC) assay was conducted according to the method previously reported by Re et al. [28]. Trolox was used as the standard, and the results are described as µmol Trolox/g DW.

The total phenolic content (TPC) was detected using the method reported by Singleton et al. [29]. In brief, a 500 µL sample was added into 2.5 mL Folin–Ciocalteu reagent (0.2 mol/L) to react for 4 min at room temperature in the dark. Subsequently, 2 mL of saturated sodium carbonate solution (75 g/L) was added into the mixture which was measured for absorbance at 760 nm after 2 h of incubation at room temperature in the dark. Gallic acid was applied as a standard, and the results are presented as mg gallic acid equivalent (GAE)/g DW.

### 2.9. Measurement of Phytochemicals in Teas

The phytochemicals in tea infusions were detected by HPLC according to a previous report in the literature with slight alterations [30]. The measurement instrument consisted of an HPLC pump separation module (Waters 1525, Milford, MA, USA), a photodiode array detector (Waters 2996, USA), and an Agilent Zorbax Eclipse XDB-C18 column (4.6 × 250 mm, 5 µm, Santa Clara, CA, USA). Briefly, the separation was carried out at 35 °C, and the flow rate was set at 1.0 mL/min. The mobile phases were composed of 0.1% formic acid (solution A) and methanol (solution B). The elution gradient was performed as follows: 0 min (2% B), 10 min (17% B), 15 min (19% B), 20 min (22% B), 40 min (47% B), 50 min (50% B), 60 min (58% B), 70 min (2% B), 70.1 min (2% B), and 75 min (2% B). Target phytochemicals in teas were identified according to the retention time and spectra of the standards and quantified based on the peak area under the maximum absorption wavelength. The results were recorded as mg/g DW of tea.

### 2.10. Statistical Analysis

The statistical analysis was performed using SPSS 23.0 (IBM SPSS Statistics, IBM Corp, Somers, NY, USA). The statistical significance was tested by one-way analysis of variance (ANOVA) and post-hoc least significant difference (LSD) test. Statistical significance was defined at *p* < 0.05.

## 3. Results and Discussion

### 3.1. Effects of Tea on ALT, AST, and ALP in Sera

**Figure 1:**
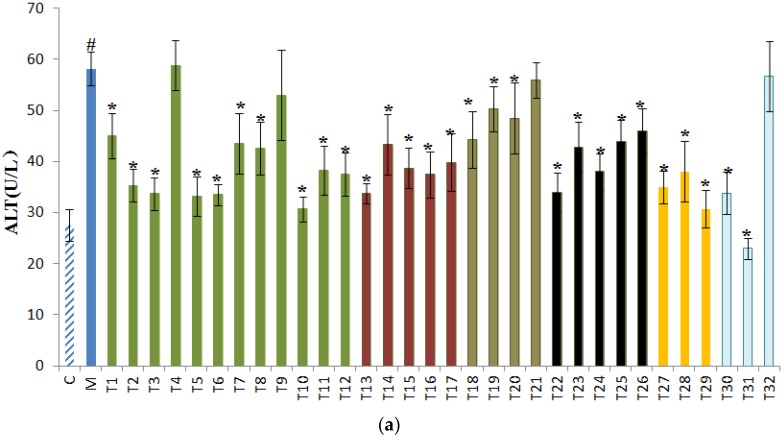

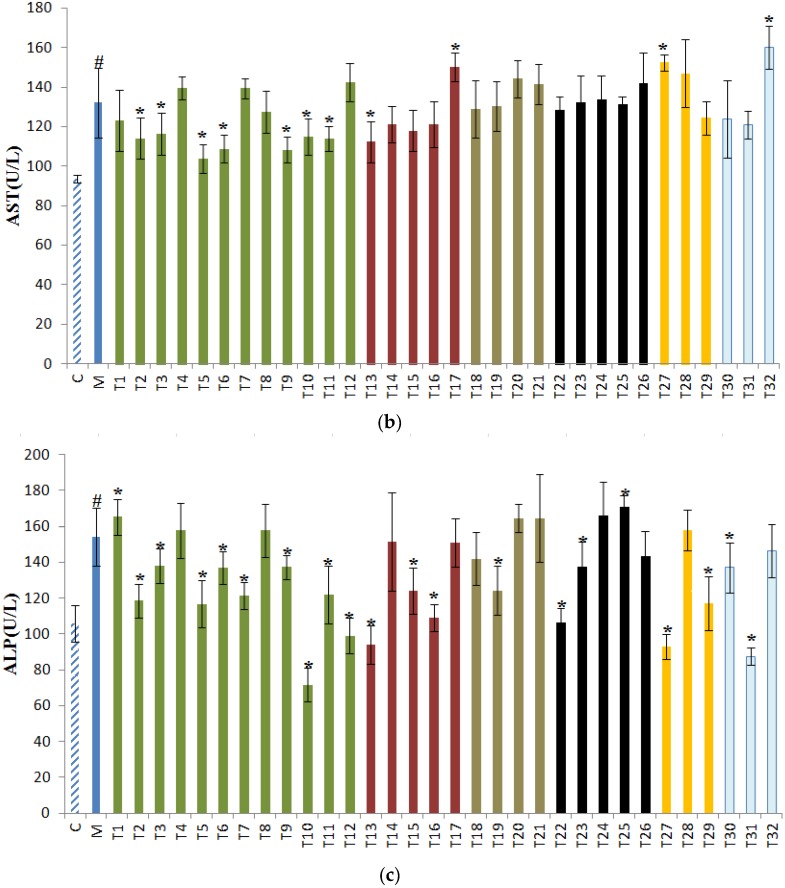
The effects of 32 teas on serum (**a**) alanine transaminase (ALT); (**b**) aspartate transaminase (AST); (**c**) alkaline phosphatase (ALP) levels. ^#^ *p* < 0.05, the model group versus the control group. * *p* < 0.05, the treatment group versus the model group. C, the control group; M, the model group; T1, Chaoqing Green Tea; T2, selenium-enriched Chaoqing Green Tea; T3, Enshi Yulu Tea; T4, selenium-enriched Matcha; T5, Xihu Longjing Tea; T6, Matcha; T7, Fried Green Tea; T8, Taiping Houkui Tea; T9, Dianqing Tea; T10, Liping Xiang Tea; T11, Jieyang Chaoqing Tea; T12, Fenggang zinc-selenium-enriched Tea; T13, Yihong Tea; T14, selenium-enriched Black Tea; T15, Dianhong Tea; T16, Lapsang Souchong Tea; T17, Yingde Black Tea; T18, Tieguanyin Tea; T19, Wuyi Narcissus Tea; T20, Fenghuang Danzong Tea; T21, Fenghuang Narcissus Tea; T22, Fu Brick Tea; T23, Qing Brick Tea; T24, Pu-erh Tea; T25, Liupao Tea; T26, Black Fu Brick Tea; T27, Yuan’an Luyuan Tea; T28, Mengding Huangya Tea; T29, Huoshan Yellow Tea; T30, White Peony Tea; T31, Gongmei White Tea; T32, Shoumei White Tea.

The levels of serum ALT, AST, and ALP were detected to assess the liver injury degree. As shown in Figure 1, alcohol caused the elevation of serum ALT, AST, and ALP levels of model group mice compared with the control group, indicating the existence of liver damage. In Figure 1a, 28 out of 32 teas significantly (*p* < 0.05) reduced the ALT level. Gongmei White Tea (T31) showed the strongest ALT lowering activity, followed by Liping Xiang Tea (T10), Huoshan Yellow Tea (T29), Xihu Longjing Tea (T5), and Matcha (T6). Based on Figure 1b, selenium-enriched Chaoqing Green Tea (T2), Enshi Yulu Tea (T3), Xihu Longjing Tea (T5), Matcha (T6), Dianqing Tea (T9), Liping Xiang Tea (T10), Jieyang Chaoqing Tea (T11), and Yihong Tea (T13) significantly (*p* < 0.05) decreased serum AST level, but Yingde Black Tea (T17), Yuan’an Luyuan Tea (T27), and Shoumei White Tea (T32) increased the serum AST level. According to Figure 1c, 19 teas significantly reduced the serum ALP level, and Liping Xiang Tea (T10), Gongmei White Tea (T31), Yuan’an Luyuan Tea (T27), Yihong Tea (T13), and Fenggang zinc-selenium-enriched tea (T12) were the top five teas that could lower the ALP level. However, Chaoqing Green Tea (T1) and Liupao Tea (T25) consumption led to an increase in serum ALP levels. Similar complicated results were also observed in a previous report which studied the hepatoprotective effects of 20 herbal infusions, teas, and carbonated beverages on alcohol-induced liver injury [31].

Both ALT and AST are important aminopherases in hepatocyte cytoplasm. The plasma ALT and AST levels can be increased when hepatocytes are damaged [32]. The alanine aminopherase (ALT) is a more specific biomarker of liver injury than AST, because ALT is mainly expressed in the liver, while AST can be detected in the liver, skeletal muscle, and cardiac muscle [31]. Consumption of alcohol can induce the liver damage with increased serum ALT and AST levels [33]. In our study, the AST and ALT levels of the model group were higher than those of the control group. The treatment of 32 tea extracts showed different effects on the increased serum AST and ALT levels that were induced by alcohol which might be due to the differences in the antioxidant profiles of the teas. The results showed that Gongmei White Tea (T31), Liping Xiang Tea (T10), Huoshan Yellow Tea (T29), Xihu Longjing Tea (T5), and Matcha (T6) remarkably reduced the serum ALT level by approximately 50% compared with the model group, indicating their notable protective effects against alcohol-induced liver injury. However, most teas showed no evident influence on serum AST level. Surprisingly, treatment of Yingde Black Tea (T17), Yuan’an Luyuan Tea (T27), and Shoumei White Tea (T32) further elevated serum AST level, suggesting that these three teas might aggravate the development of liver injury induced by alcohol. Therefore, the consumption of these three teas is not suggested after drinking alcohol.

The alkaline phosphatase (ALP) is usually elevated in patients and animals with biliary tract dysfunction [34]. In our study, the ALP level of the model group was elevated compared with the control group. Most teas restored the serum ALP level which indicated that these teas could improve the function of the biliary tract. However, intake of Chaoqing Green Tea (T1) and Liupao Tea (T25) caused a raise in serum ALP levels, indicating that these two teas should not be drunk after the consumption of alcohol. In short, most teas at a dose of 400 mg/kg could improve liver function against alcohol by reducing the levels of ALT, AST, and ALP.

### 3.2. Effects of Tea on TG, TBIL, and TP in Sera

**Figure 2:**
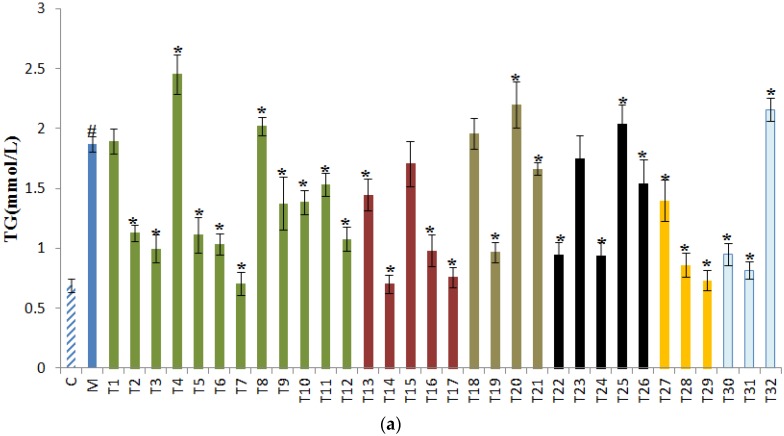

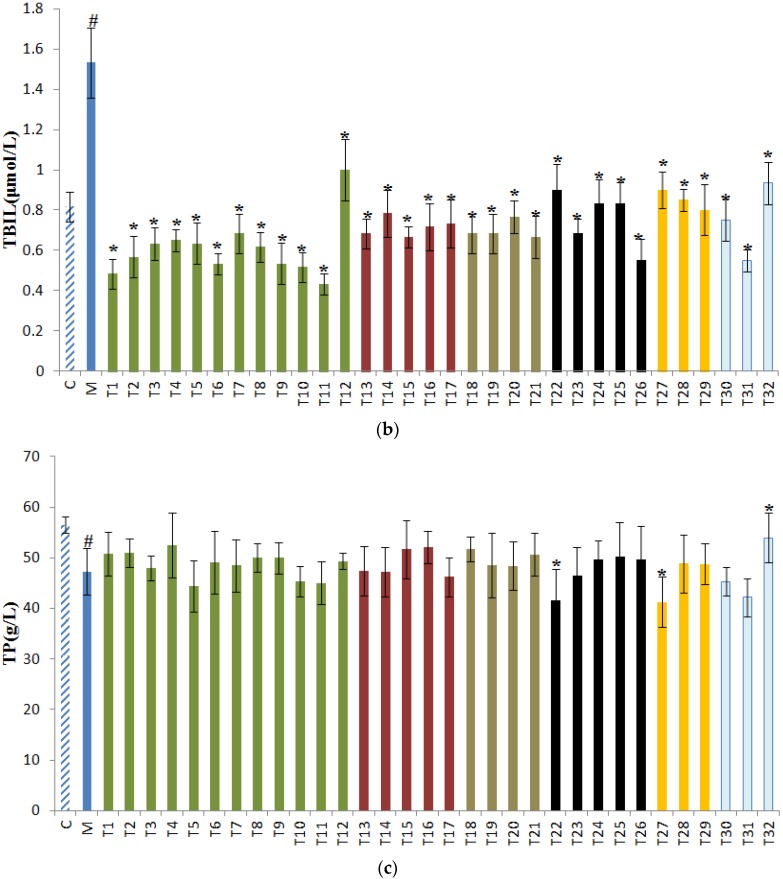
The effects of 32 teas on serum (**a**) triacylglycerol (TG); (**b**) total bilirubin (TBIL); (**c**) total protein (TP) levels. ^#^ *p* < 0.05, the model group versus the control group. * *p* < 0.05, the treatment group versus the model group. C, the control group; M, the model group; T1, Chaoqing Green Tea; T2, selenium-enriched Chaoqing Green Tea; T3, Enshi Yulu Tea; T4, selenium-enriched Matcha; T5, Xihu Longjing Tea; T6, Matcha; T7, Fried Green Tea; T8, Taiping Houkui Tea; T9, Dianqing Tea; T10, Liping Xiang Tea; T11, Jieyang Chaoqing Tea; T12, Fenggang zinc-selenium-enriched Tea; T13, Yihong Tea; T14, selenium-enriched Black Tea; T15, Dianhong Tea; T16, Lapsang Souchong Tea; T17, Yingde Black Tea; T18, Tieguanyin Tea; T19, Wuyi Narcissus Tea; T20, Fenghuang Danzong Tea; T21, Fenghuang Narcissus Tea; T22, Fu Brick Tea; T23, Qing Brick Tea; T24, Pu-erh Tea; T25, Liupao Tea; T26, Black Fu Brick Tea; T27, Yuan’an Luyuan Tea; T28, Mengding Huangya Tea; T29, Huoshan Yellow Tea; T30, White Peony Tea; T31, Gongmei White Tea; T32, Shoumei White Tea.

Elevated TG and TBIL as well as decreased TP levels are biomarkers of alcohol-induced liver injury. In Figure 2, compared with the control group, alcohol consumption significantly increased the serum TG and TBIL levels and decreased the serum TP level. According to Figure 2a, most teas greatly reduced the serum TG level (*p* < 0.05), and selenium-enriched Black Tea (T14), Fried Green Tea (T7), Huoshan Yellow Tea (T29), Yingde Black Tea (T17), and Gongmei White Tea (T31) were the top five teas for decreasing serum TG levels. However, selenium-enriched Matcha (T4), Fenghuang Danzong Tea (T20), Shoumei White Tea (T32), Liupao Tea (T25), and Taiping Houkui Tea (T8) stimulated the serum TG. As shown in Figure 2b, administration of all 32 teas effectively lowered serum TBIL level, and the top five teas were Jieyang Chaoqing Tea (T11), Chaoqing Green Tea (T1), Liping Xiang Tea (T10), Matcha (T6), and Dianqing Tea (T9). Decreased serum TP levels is another feature of alcohol-induced liver injury. According to Figure 2c, most teas showed no evident influences on serum TP levels, and only Shoumei White Tea (T32) elevated the serum TP level, while Fu Brick Tea (T22) and Yuan’an Luyuan Tea (T14) downregulated the serum TP level.

Long-term alcohol consumption leads to the accumulation of TG in the liver which can be delivered into the blood [35]. Our results indicated that most teas improved lipid metabolism against alcohol-induced liver injury. In contrast, treatment of selenium-enriched Matcha (T4), Fenghuang Danzong Tea (T20), Shoumei White Tea (T32), Liupao Tea (T25), and Taiping Houkui Tea (T8) boosted the serum TG level, suggesting that these teas aggravated the abnormality of lipids induced by alcohol. These five teas may be not suitable for drinking after alcohol intake.

Bilirubin, the product of the enzymatic degradation of heme, can be conjugated with glucuronic acid into a water-soluble compound. When biliary excretion is damaged, bilirubin can accumulate in the plasma [36]. Alcohol consumption can elevate the serum TBIL level, indicating the function impairment of the liver. Treatment of all 32 teas significantly reduced the elevated TBIL level induced by alcohol, and green tea showed the strongest TBIL lowering activity among the six tea categories. The TBIL-reducing effects of tea were also reported in a previous study [37].

Albumin, produced by the hepatocyte, is the main protein in sera and has an essential role in the metabolism and maintenance of oncotic pressure [38]. A reduced serum TP level indicates the impairment of hepatocyte. Alcohol consumption leads to a reduced serum TP level. Twenty-nine teas showed no effects on altering the TP level, while the Shoumei White Tea (T32) increased the TP level. However, Fu Brick Tea (T22) and Yuan’an Luyuan Tea (T27) might exacerbate the liver cell injury induced by alcohol, as they could reduce the TP level further.

### 3.3. Effects of 32 Teas on Antioxidant Biochemical Markers in the Liver

**Figure 3:**
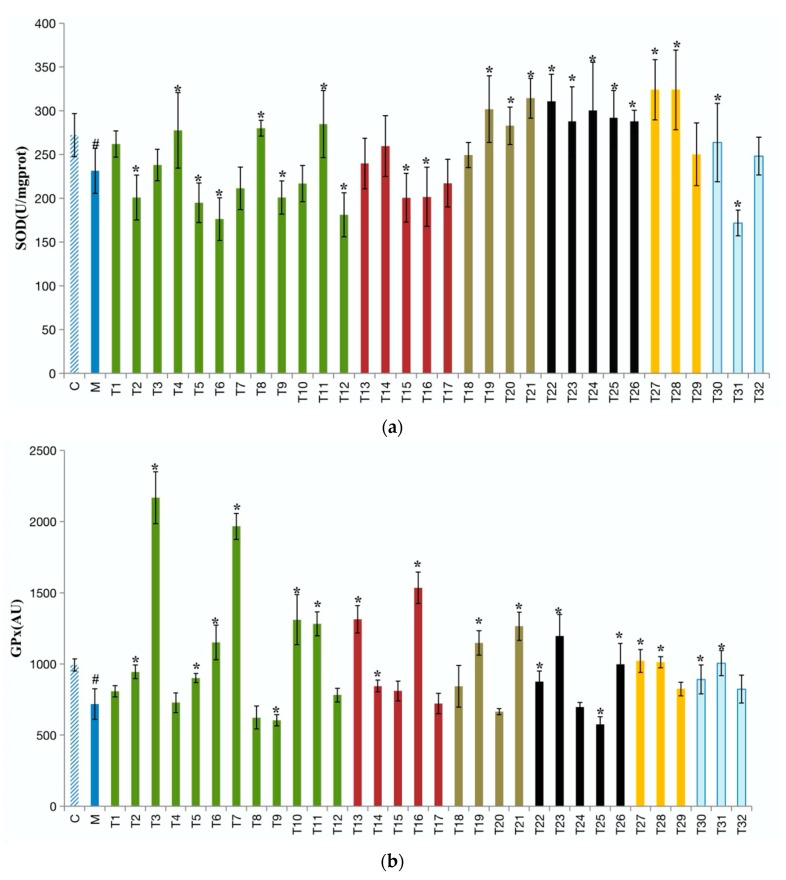

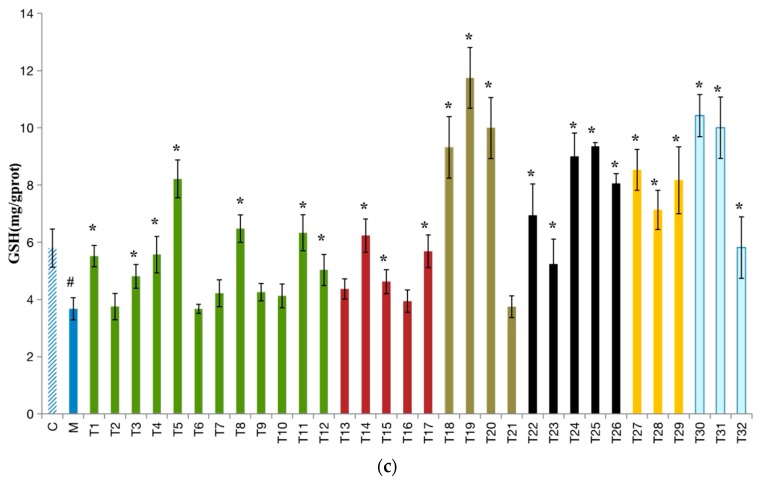
The effects of 32 teas on liver (**a**) superoxide dismutase (SOD); (**b**) glutathione peroxidase (GPx); (**c**) glutathione (GSH) levels. ^#^ *p* < 0.05, the model group versus the control group. * *p* < 0.05, the treatment group versus the model group. C, the control group; M, the model group; T1, Chaoqing Green Tea; T2, selenium-enriched Chaoqing Green Tea; T3, Enshi Yulu Tea; T4, selenium-enriched Matcha; T5, Xihu Longjing Tea; T6, Matcha; T7, Fried Green Tea; T8, Taiping Houkui Tea; T9, Dianqing Tea; T10, Liping Xiang Tea; T11, Jieyang Chaoqing Tea; T12, Fenggang zinc-selenium-enriched Tea; T13, Yihong Tea; T14, selenium-enriched Black Tea; T15, Dianhong Tea; T16, Lapsang Souchong Tea; T17, Yingde Black Tea; T18, Tieguanyin Tea; T19, Wuyi Narcissus Tea; T20, Fenghuang Danzong Tea; T21, Fenghuang Narcissus Tea; T22, Fu Brick Tea; T23, Qing Brick Tea; T24, Pu-erh Tea; T25, Liupao Tea; T26, Black Fu Brick Tea; T27, Yuan’an Luyuan Tea; T28, Mengding Huangya Tea; T29, Huoshan Yellow Tea; T30, White Peony Tea; T31, Gongmei White Tea; T32, Shoumei White Tea.

The antioxidant system includes enzymatic antioxidants, such as SOD and GPx, and non-enzymatic antioxidants, such as GSH, representing the main defense system against ROS in the liver [39]. It has been reported that the activities of SOD and GPx and the content of GSH were inhibited in alcoholics and laboratory animals [40]. In this study, consumption of alcohol significantly reduced the liver SOD and GPx activities and GSH levels compared with the control group. As shown in Figure 3a, around half of 32 teas significantly restored the SOD level, and the top five teas were Mengding Huangya Tea (T28), Yuan’an Luyuan Tea (T27), Fenghuang Narcissus Tea (T21), Black Fu Brick Tea (T26), and Wuyi Narcissus Tea (T19). In Figure 3b, 19 of the tested 32 teas significantly elevated the GPx level, and Enshi Yulu Tea (T3), Fried Green Tea (T7), Lapsang Souchong Tea (T16), Yihong Tea (T13), and Liping Xiang Tea (T10) were the top five teas in enhancing the GPx level. Only Dianqing Tea (T9) and Liupao Tea (T25) lowered the GPx level further. According to Figure 3c, most teas restored the downregulated GSH level induced by alcohol, and Wuyi Narcissus Tea (T19), White Peony Tea (T30), Gongmei White Tea(T31), Fenghuang Danzong Tea (T20), and Liupao Tea (T25) showed strong activities in elevating the GSH level by 200% compared with the model group.

The superoxide dismutase (SOD) can convert superoxide anion to a less reactive hydrogen peroxide. The inhibition of SOD activity may cause the accumulation of superoxide anion which can lead to adverse effects such as damage of the cell membrane integrity and functional disorder [41]. In the literature, the effects of plant antioxidants on the activity of SOD is complicated, because the activity of SOD may increase, decrease, or induce no change at all [4,42]. This may be due to the differences in antioxidant compounds or the bioavailabilities. The antioxidant profiles of 32 teas were very different, probably due to the differences in their production process [43]. Among the 32 tested teas, 14 teas significantly enhanced the activity of SOD, indicating that these teas can defend the liver against oxidative stress induced by alcohol consumption. However, Gongmei White Tea (T31), Matcha (T6), Fenggang zinc-selenium-enriched Tea (T12), Xihu Longjing Tea (T5), Dianhong Tea (T15), Dianqing Tea (T9), selenium-enriched Chaoqing Green tea (T2), and Lapsang Souchong Tea (T16) reduced the SOD activity which may be related to the deteriorative liver injury.

The glutathione peroxidase (GPx), an important antioxidant enzyme located in both the cytoplasm and mitochondria, can protect membrane lipids, proteins, and nucleic acids against free radical damage [44]. Most teas significantly enhanced the GPx level, indicating that they can protect alcohol-induced oxidative liver injury. Additionally, GSH is a main non-enzymatic antioxidant in mammals. The glutathione (GSH) is the substrate of GPx and can be converted into oxidized GSH by GPx [45]. Overall, all teas elevated the GSH level, while green tea and black tea elevated the GSH level less than the other four tea categories.

Generally, most teas showed strong in vivo antioxidant activities. Tea contains many natural antioxidants, such as catechins, caffeine, theaflavin, gallic acid, chlorogenic acid, ellagic acid, and kaempferol-3-*O*-glucoside, as reported in our previous studies [15,16].

### 3.4. Effects of 32 Teas on Lipid Peroxidation Levels in the Liver

**Figure 4:**
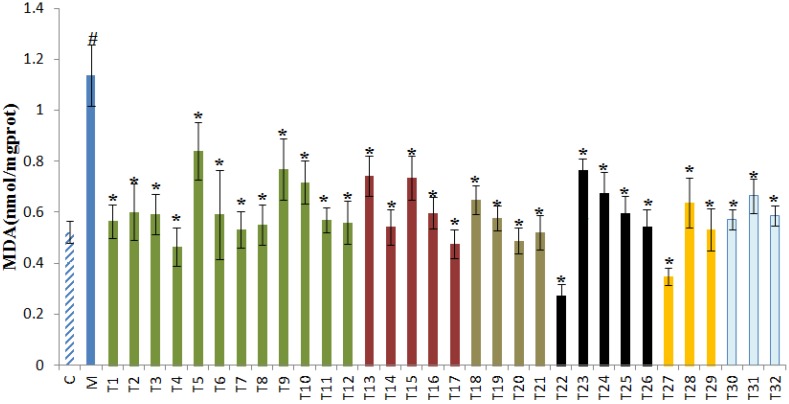
The effects of 32 teas on liver malondialdehyde (MDA) level. ^#^ *p* < 0.05, the model group versus the control group. * *p* < 0.05, the treatment group versus the model group. C, the control group; M, the model group. T1, Chaoqing Green Tea; T2, selenium-enriched Chaoqing Green Tea; T3, Enshi Yulu Tea; T4, selenium-enriched Matcha; T5, Xihu Longjing Tea; T6, Matcha; T7, Fried Green Tea; T8, Taiping Houkui Tea; T9, Dianqing Tea; T10, Liping Xiang Tea; T11, Jieyang Chaoqing Tea; T12, Fenggang zinc-selenium-enriched Tea; T13, Yihong Tea; T14, selenium-enriched Black Tea; T15, Dianhong Tea; T16, Lapsang Souchong Tea; T17, Yingde Black Tea; T18, Tieguanyin Tea; T19, Wuyi Narcissus Tea; T20, Fenghuang Danzong Tea; T21, Fenghuang Narcissus Tea; T22, Fu Brick Tea; T23, Qing Brick Tea; T24, Pu-erh Tea; T25, Liupao Tea; T26, Black Fu Brick Tea; T27, Yuan’an Luyuan Tea; T28, Mengding Huangya Tea; T29, Huoshan Yellow Tea; T30, White Peony Tea; T31, Gongmei White Tea; T32, Shoumei White Tea.

Oxidative stress plays a key role in the development of liver injury induced by alcohol. Malondialdehyde (MDA), an end product of lipid peroxidation induced by ROS, represents the peroxidative degree of tissue [44]. Figure 4 shows the results of hepatic lipid peroxidation level. Compared with the control group, the level of lipid peroxidation significantly increased in the model group, indirectly reflecting the damage of liver cell. All treatments of teas greatly attenuated the MDA level which indicates that teas can ameliorate the peroxidation of hepatocytes caused by excessive alcohol intake. The results are consistent with the results of the histopathological evaluation. Tea is rich in polyphenols, such as catechins, quercetin, and theaflavin, which have been proposed to attenuate the lipid peroxidation [46,47]. Therefore, these tea polyphenols might be responsible for reducing MDA levels.

### 3.5. Histopathological Evaluation

**Figure 5:**
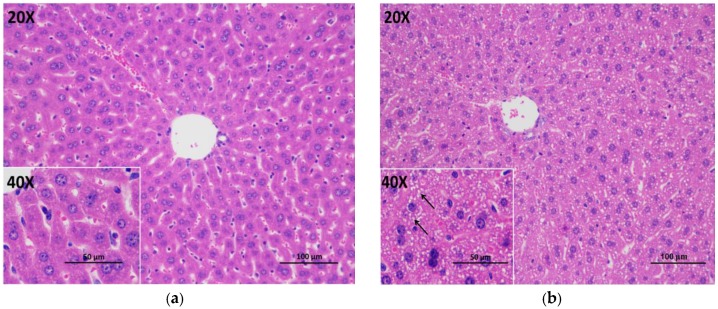

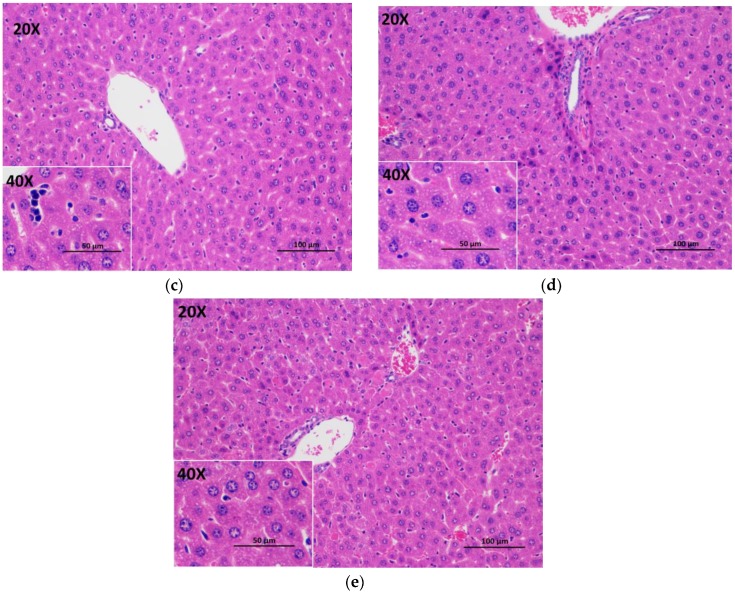
The photomicrographs of liver sections stained by hematoxylin–eosin: (**a**) control group; (**b**) model group; (**c**) Black Fu Brick Tea; (**d**) Wuyi Narcissus Tea; (**e**) Pu-erh Tea. Arrows indicate lipid drops.

Figure 5 showed the protective role of tea against alcohol-induced liver injury using a histopathological assay. As shown in Figure 5a, there were no visible lesions in the control group. Compared with the control group, alcohol consumption led to obvious pathologic alterations with lipid drops depositing on the cytoplasm in the model group. Tea treatment remarkably reduced the accumulation of lipid drops and attenuated the liver injury induced by the alcohol intake (Figure 5c–e). The result of this study was in agreement with a previous research reporting the protective effect of green tea against alcohol-induced liver histopathological alterations [48].

### 3.6. Systematic Cluster of SOD and MDA Values of 32 Teas

**Figure 6:**
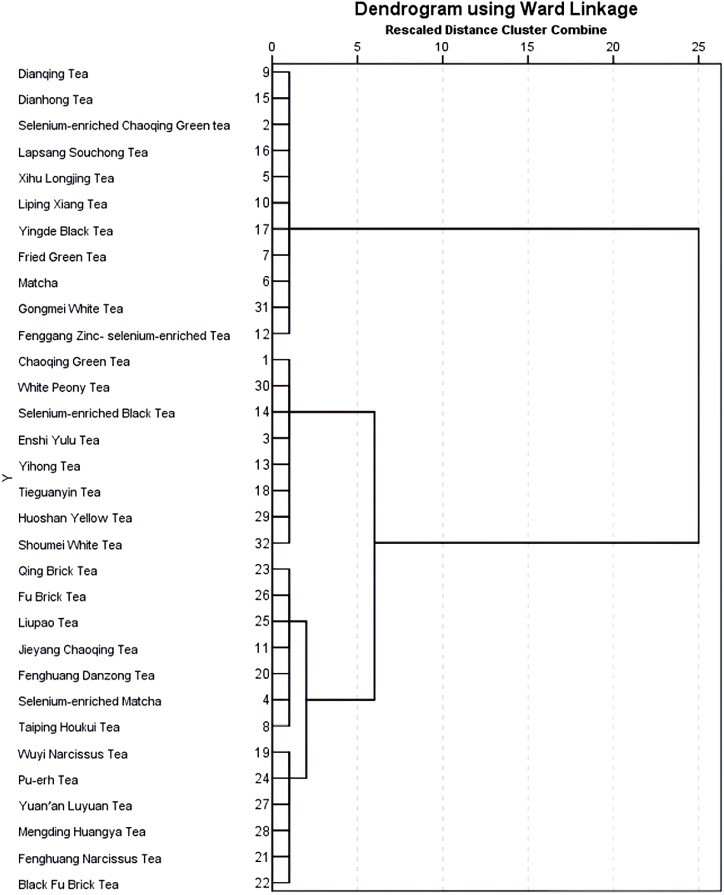
Dendrogram using Ward linkage from the systematic cluster analysis of 32 Chinese teas.

To compare the in vivo antioxidant capacities of six tea categories, a systematic cluster analysis of 32 teas was carried out with cluster numbers 2 to 6 based on the SOD and MDA values. The systematic cluster analysis of the in vitro antioxidant capacities of six tea categories was reported in a previous study [15]. The results of the systematic cluster analysis are presented in Figure 6. Subsequently, the outcomes of cluster number 3 were analyzed employing online analytical processing (OLAP) and one-way variance analysis (ANOVA), and the results are displayed in Table 2. Cluster 1 comprised eight teas, including two green teas, two black teas, one oolong tea, one yellow tea, and two white teas with the medium SOD and MDA values of 251.35 ± 9.71 U/mgprot and 0.60 ± 0.07 mol/mgprot. Additionally, cluster 2 included 11 teas which were seven green teas, three black teas, and one white tea. Cluster 2 had a relatively low SOD value of 197.57 ± 15.5 U/mgprot and the highest MDA value of 0.64 ± 0.11 nmol/mgprot. Moreover, cluster 3 contained all five dark teas, three green teas, three oolong teas, and two yellow tea with relatively high SOD values of 297.54 ± 16.24 U/mgprot and the lowest MDA value of 0.54 ± 0.13 nmol/mgprot. According to the ANOVA results, all the differences were significant among the 3 clusters regarding the SOD and MDA values (*p* < 0.05).

Cluster 1 contained eight teas. These teas showed medium in vivo antioxidant capacity. Besides, the cluster 2 possessed relatively low SOD and relatively high MDA values, which suggested the in vivo antioxidant activities of these teas were low. Cluster 2 contained 11 teas, seven of which were green teas. Green teas usually had remarkably high in vitro antioxidant capacities [15,16]. But these green teas showed relatively low in vivo antioxidant capacities in this study. The low bioavailability of green tea catechins might contribute to their low in vivo antioxidant capacity [30]. In addition, cluster 3 included all five dark teas which had the highest SOD values and the lowest MDA values. Dark tea is reported to possess less in vitro antioxidant capacity as their fermented degree is very high [49], but they showed stronger in vivo antioxidant capacity in this study. The fermentation products of dark tea, such as theabrownin and theophylline, might contribute to their high in vivo antioxidant capacity [49,50]. Therefore, the in vivo antioxidant capacity of teas might be opposite to in vitro antioxidant capacity which should be further investigated in the future.

### 3.7. Phenolic Compounds in Teas

**Figure 7:**
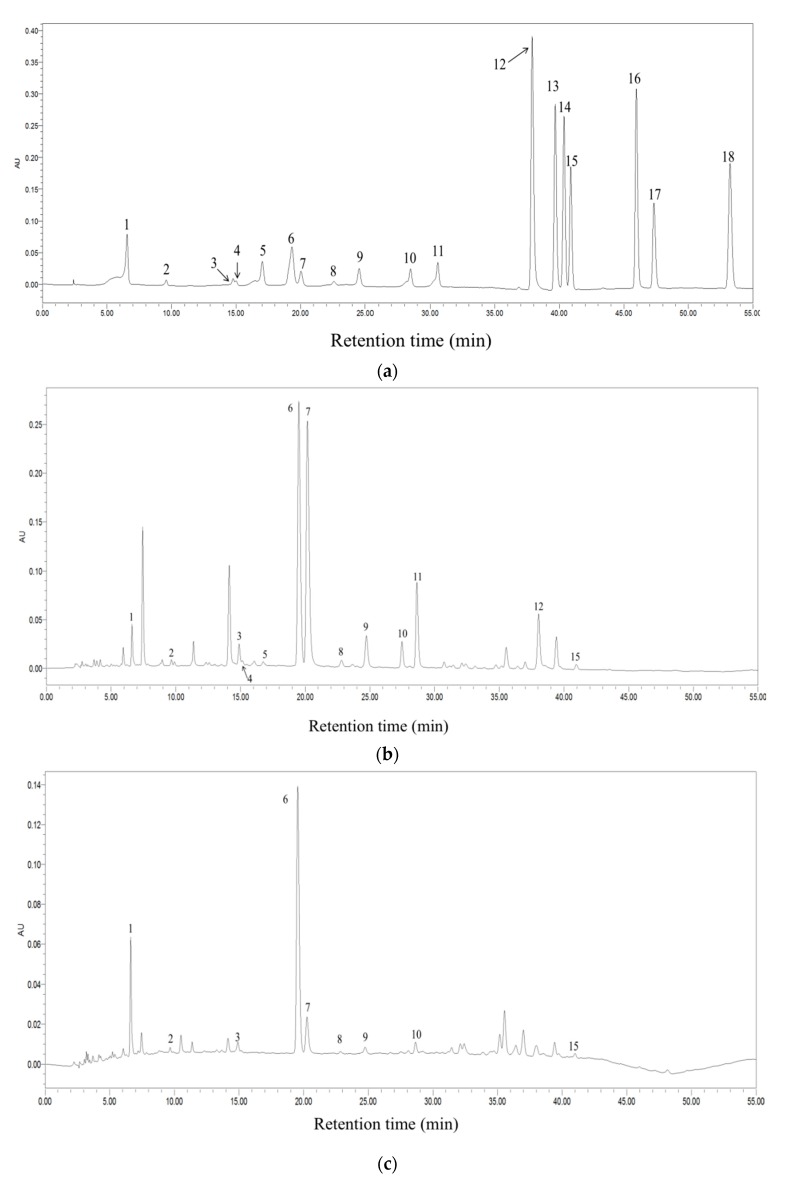
The HPLC chromatograms of the standard compounds (**a**), Fenggang zinc-selenium-enriched Tea (**b**), and Black Fu Brick Tea (**c**) under 254 nm. 1, gallic acid; 2, gallocatechin; 3, epigallocatechin; 4, catechin; 5, chlorogenic acid; 6, caffeine; 7, epigallocatechin gallate; 8, epicatechin; 9, gallocatechin gallate; 10, epicatechin gallate; 11, catechin gallate; 12, ellagic acid; 13, myricetin; 14, quercitrin; 15, astragalin; 16, quercetin; 17, theaflavin; 18, kaempferol.

The phenolic compounds in different tea extracts were detected by HPLC. The chromatograms of the standards, Fenggang zinc-selenium-enriched tea, and Black Fu Brick Tea at 254 nm are presented in Figure 7. The phytochemical contents, ferric-reducing antioxidant power (FRAP), Trolox equivalent antioxidant capacity (TEAC), and total phenolic content in 14 teas are presented in Table 3 and Table 4. The results of the other 18 teas can be found in our previous paper on the in vitro antioxidant activities of teas [16]. A total of 16 phytochemicals were identified and quantified, including GC, EGC, C, EGCG, EC, GCG, ECG, and CG, gallic acid, chlorogenic acid, caffeine, ellagic acid, quercitrin, astragalin, quercetin, and theaflavin. Specifically, gallic acid and caffeine were detected in all 32 Chinese teas. In addition, EC (31), EGCG (28), ECG (26), EGC (24), GC (22), EA (22), GCG (21) were found in most teas.

The most abundant phytochemicals identified in these teas were catechins which accounted for up to 17.05% DW of tea leaves, but the contents of catechins greatly differed among teas. Among eight catechins, EGCG was the richest catechin in teas, ranging from 1.02 ± 0.07 to 56.30 ± 4.59 mg/g DW, and green tea and oolong tea had higher EGCG contents than black tea and dark tea. In addition, EC was detected in the majorities of these teas with a range of 0.77 ± 0.02 to 16.40 ± 1.34 mg/g DW. The 26 teas were found with ECG contents ranging from 2.37 ± 0.22 to 47.78 ± 3.39 mg/g DW. The contents of catechin and CG were both low in tested teas, with values ranging from 1.01 ± 0.08 to 7.05 ± 0.35 mg/g DW and from 0.43 ± 0.03 to 3.01 ± 0.19 mg/g DW, respectively, and was only detected in 11 and 10 teas, respectively.

Besides catechins, gallic acid was found in all tested teas, and the contents varied from 0.29 ± 0.02 to 4.44 ± 0.21 mg/g DW. Selenium-enriched Black Tea, Yihong Tea, Fenghuang Danzong Tea, Fu Brick Tea, and Huoshan Yellow Tea were the top five teas possessing the highest gallic acid contents. Additionally, all tested teas were found with high contents of caffeine, with a range of 12.36 ± 1.18 to 40.22 ± 3.17 mg/g DW. The top five teas with the highest caffeine contents were Yuan’an Luyuan Tea (40.22 ± 3.17 mg/g DW), Enshi Yulu Tea (38.14 ± 3.01 mg/g DW), Dianqing Tea (38.01 ± 2.46 mg/g DW), Fenghuang Danzong Tea (37.95 ± 2.84 mg/g DW), and Xihu Longjing Tea (36.65 ± 3.28 mg/g DW). Furthermore, certain teas contained astragalin but with low values, ranging from 0.30 ± 0.01 to 2.11 ± 0.13 mg/g DW. Moreover, chlorogenic acid, quercitrin, ellagic acid, quercetin, and theaflavin were also found in some teas, while theaflavin was only detected in tested black teas.

Generally speaking, catechins were most abundant in green tea and much less in black tea and dark tea. This may be associated with the fermentation degrees, as green tea was non-fermented, while black tea and dark tea were highly fermented. It was reported that the differences of manufacture procedure of these six tea types were the major reasons to cause the alterations of tea catechin profiles [43]. In addition, as the degrees of fermentation increased, the contents of gallic acid in teas also increased. On the other hand, the caffeine contents in teas were relatively stable.

The FRAP value is an important index of antioxidant capacity. The FRAP value was used to indicate the ability of tea in reducing ferric ions to ferrous ions [15]. The FRAP value of these 32 teas ranged from 530.13 ± 23.44 to 4647.47 ± 57.87 µmol Fe^2+^/g DW. The top five teas possessing the highest FRAP value were Dianqing Tea (4647.47 ± 57.87 µmol Fe^2+^/g DW), Yuan’an Luyuan Tea (4088.80 ± 118.39 µmol Fe^2+^/g DW), Fenggang Zinc-selenium-enriched Tea (3891.20 ± 64.65 µmol Fe^2+^/g DW), Liping Xiang Tea (3891.20 ± 75.60 µmol Fe^2+^/g DW), and Xihu Longjing Tea (3872.80 ± 38.16). The TEAC assay indicates the antioxidant capacity of scavenging free radicals. The TEAC values of teas ranged from 381.3 ± 18.70 to 2532.41 ± 50.18 µmol Trolox/g DW. Among these 32 teas, Dianqing Tea (2532.41 ± 50.18 µmol Trolox/g DW), Fenggang zinc-selenium-enriched tea (2162.40 ± 24.51 µmol Trolox/g DW), selenium-enriched Chaoqing Green Tea (1960.46 ± 23.69 µmol Trolox/g DW), Xihu Longjing Tea (1935.89 ± 26.32 µmol Trolox/g DW), and Yuan’an Luyuan Tea (1835.52 ± 19.60 µmol Trolox/g DW) were the top five teas with the highest TEAC values. The TPC of these 32 teas differed from 37.23 ± 0.28 to 252.65 ± 4.74 mg GAE/g DW. Dianqing Tea, Fenggang Zinc-selenium-enriched Tea, Yuan’an Luyuan Tea, Liping Xiang Tea, and Xihu Longjing Tea were the top five teas with the highest TPC values of 252.65 ± 4.74, 251.65 ± 4.43, 220.08 ± 1.75, 219.99 ± 1.42, and 218.46 ± 8.82 mg GAE/g DW, respectively. Overall, the antioxidant capacity and total phenolics of 32 teas greatly varied, and green tea possessed the highest in vitro antioxidant capacity and total phenolics among the six tea categories.

**Figure 8:**
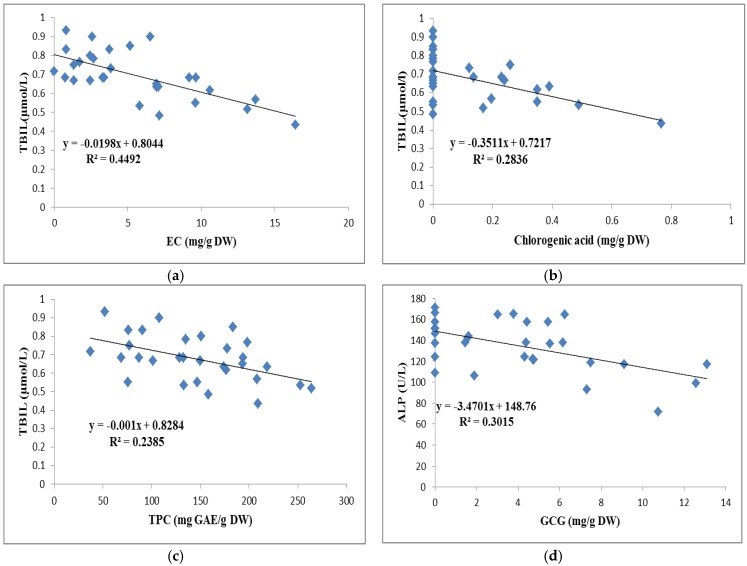
Correlations between EC and TBIL (**a**), chlorogenic acid and TBIL (**b**), TPC and TBIL (**c**), GCG and ALP (**d**). EC, epicatechin; TBIL, total bilirubin level; TPC, total phenolic content; GCG, gallocatechin gallate; ALP, alkaline phosphatase.

In order to study the relationships between the phytochemicals and the bioactivities of teas, we analyzed the correlations among 10 biomarkers and 16 phytochemicals as well as FRAP, TEAC, and TPC. The results of the correlation analysis indicated that EC, chlorogenic acid, and TPC had moderate negative correlations with TBIL level, and the *R^2^* values were 0.4492, 0.2836, and 0.2385 (*p* < 0.05), respectively (Figure 8). These results suggest that EC, chlorogenic acid, and TPC in teas, at least partly, contributed to the TBIL lowering ability. Besides, the GCG content was also found to negatively relate to the ALP value, with an *R^2^* value of 0.3015 (*p* < 0. 05) which implies that the GCG of teas play a role in reducing the ALP value against alcohol-induced liver injuries. There were no significant correlations between the rest of phytochemicals and in vivo biomarkers.

## 4. Conclusions

In this study, the in vivo antioxidant and hepatoprotective effects of 32 Chinese teas were evaluated and compared. The results indicated that these teas possessed very different in vivo antioxidant and hepatoprotective activities. Generally, most teas could protect against liver injury induced by alcohol by lowering the serum ALT, AST, ALP, TBIL, and TG levels, enhancing antioxidant enzyme activities, and attenuating lipid peroxidation. Among them, Black Fu Brick Tea, Fenghuang Narcissus Tea, Wuyi Narcissus Tea, Pu-erh Tea, and Qing Brick Tea showed the strongest hepatoprotective and in vivo antioxidant activities. These teas can be consumed after alcohol intake to attenuate the adverse effects of ethanol and can also be utilized to prevent and treat oxidative stress-related diseases. Jieyang Chaoqing Tea (green tea), selenium-enriched Black Tea (black tea), Wuyi Narcissus Tea (oolong tea), Black Fu Brick Tea (dark tea), Huoshan Yellow Tea (yellow tea), and White Peony Tea (white tea) showed the strongest hepatoprotective and in vivo antioxidant activities in their corresponding categories. Thus, people can select one of these six teas according to their preferred tea category which should have better health benefits at the same time. On the other hand, several teas were not suggested to consume after alcohol intake, such as Yingde Black Tea, Yuan’an Luyuan Tea, Shoumei White Tea, Chaoqing Green Tea, and Liupao Tea, because these teas might deteriorate the liver lesion induced by alcohol. In addition, catechins and caffeine were mostly found in tested teas. The TPC and the contents of epicatechin, gallocatechin gallate, and chlorogenic acid were moderately associated with the antioxidant and hepatoprotective actions of tested teas. Besides, green tea had the highest in vitro antioxidant capacity, but possessed the lowest in vivo antioxidant capacity. Dark tea showed the lowest in vitro antioxidant capacity but exhibited the strongest in vivo antioxidant activities. Therefore, for developing tea and its bioactive compounds as food additives, such as antioxidants, it is recommended to choose green tea which possesses strong in vitro antioxidant capacity. However, if teas are used to prevent or manage alcoholic liver injuries and certain oxidative stress-related diseases, it may be better to pick dark tea because dark tea can remarkably enhance in vivo antioxidant capacity.

## Figures and Tables

**Table 1:** The information of 32 selected Chinese teas.

No.	Name	Category	Fermentation Degree	Production Place
T1	Chaoqing Green Tea	Green Tea	Unfermented	Yichang, Hubei
T2	Selenium-Enriched Chaoqing Green Tea	Green Tea	Unfermented	Enshi, Hubei
T3	Enshi Yulu Tea	Green Tea	Unfermented	Enshi, Hubei
T4	Selenium-Enriched Matcha	Green Tea	Unfermented	Enshi, Hubei
T5	Xihu Longjing Tea	Green Tea	Unfermented	Hangzhou, Zhejiang
T6	Matcha	Green Tea	Unfermented	Shaoxing, Zhejiang
T7	Fried Green Tea	Green Tea	Unfermented	Shaoxing, Zhejiang
T8	Taiping Houkui Tea	Green Tea	Unfermented	Huangshan, Anhui
T9	Dianqing Tea	Green Tea	Unfermented	Kunming, Yunnan
T10	Liping Xiang Tea	Green Tea	Unfermented	Liping, Guizhou
T11	Jieyang Chaoqing Tea	Green Tea	Unfermented	Jieyang, Guangdong
T12	Fenggang Zinc-Selenium-enriched Tea	Green Tea	Unfermented	Guiyang, Guizhou
T13	Yihong Tea	Black Tea	Deep-fermented	Yichang, Hubei
T14	Selenium-Enriched Black Tea	Black Tea	Deep-fermented	Enshi, Hubei
T15	Dianhong Tea	Black Tea	Deep-fermented	Xishuangbanna, Yunnan
T16	Lapsang Souchong Tea	Black Tea	Deep-fermented	Xiamen, Fujian
T17	Yingde Black Tea	Black Tea	Deep-fermented	Yingde, Guangdong
T18	Tieguanyin Tea	Oolong Tea	Semi-fermented	Xiamen, Fujian
T19	Wuyi Narcissus Tea	Oolong Tea	Semi-fermented	Wuyishan, Fujian
T20	Fenghuang Danzong Tea	Oolong Tea	Semi-fermented	Shantou, Guangdong
T21	Fenghuang Narcissus Tea	Oolong Tea	Semi-fermented	Shantou, Guangdong
T22	Fu Brick Tea	Dark Tea	Post-fermented	Changsha, Hunan
T23	Qing Brick Tea	Dark Tea	Post-fermented	Yichang, Hubei
T24	Pu-erh Tea	Dark Tea	Post-fermented	Pu’er, Yunnan
T25	Liupao Tea	Dark Tea	Post-fermented	Wuzhou, Guangxi
T26	Black Fu Brick Tea	Dark Tea	Post-fermented	Enshi, Hubei
T27	Yuan’an Luyuan Tea	Yellow Tea	Light-fermented	Yichang, Hubei
T28	Mengding Huangya Tea	Yellow Tea	Light-fermented	Mengdingshan, Sichuan
T29	Huoshan Yellow Tea	Yellow Tea	Light-fermented	Pu’er, Yunnan
T30	White Peony Tea	White Tea	Mild-fermented	Fuzhou, Fujian
T31	Gongmei White Tea	White Tea	Mild-fermented	Fuzhou, Fujian
T32	Shoumei White Tea	White Tea	Mild-fermented	Fuzhou, Fujian

**Table 2:** Online analytical processing (OLAP) cubes based on the systematic cluster analysis of 32 Chinese teas (cluster number 3).

Ward Method		SOD	MDA
1	Sum	2010.79	4.78
	*N*	8	8
	Mean	251.35	0.60
	SD	9.71	0.07
	% of Total Sum	25.0%	25.3%
	% of Total *N*	25.0%	25.0%
2	Sum	2173.29	7.08
	*N*	11	11
	Mean	197.57	0.64
	SD	15.5	0.11
	% of Total Sum	27.0%	37.5%
	% of Total *N*	34.4%	34.4%
3	Sum	3867.98	7.01
	*N*	13	13
	Mean	297.54	0.54
	SD	16.24	0.13
	% of Total Sum	48.0%	37.1%
	% of Total *N*	40.6%	40.6%
Total	Sum	8052.07	18.87
	*N*	32	32
	Mean	251.63	0.59
	SD	46.06	0.12
	% of Total Sum	100.0%	100.0%
	% of Total *N*	100.0%	100.0%

**Table 3:** The contents (mg/g DW) of main phytochemicals in selected teas.

**Name**	**Category**	**Gallocatechin**	**Epigallocatechin**	**Catechin**	**Epigallocatechin Gallate**	**Epicatechin**	**Gallocatechin Gallate**	**Epicatechin Gallate**	**Catechin Gallate**
Chaoqing Green Tea	Green tea	3.26 ± 0.3	8.02 ± 0.48	-	23.72 ± 1.91	7.17 ± 0.44	3.78 ± 0.27	2.64 ± 0.23	0.43 ± 0.03
Selenium-Enriched Chaoqing Green Tea	Green tea	5.24 ± 0.42	5.13 ± 0.37	3.52 ± 0.25	29.86 ± 2.46	13.70 ± 1.1	7.50 ± 0.62	24.99 ± 2.10	-
Selenium-Enriched Matcha	Green tea	3.85 ± 0.29	24.93 ± 0.51	-	27.56 ± 2.34	6.96 ± 0.46	4.41 ± 0.18	4.23 ± 0.33	0.51 ± 0.02
Matcha	Green tea	3.88 ± 0.19	11.07 ± 0.61	-	11.55 ± 1.04	3.60 ± 0.23	5.52 ± 0.25	7.64 ± 0.61	0.95 ± 0.07
Fried Green Tea	Green tea	7.22 ± 0.55	41.18 ± 2.58	1.01 ± 0.08	30.97 ± 2.93	9.64 ± 0.42	4.74 ± 0.41	16.92 ± 1.20	-
Liping Xiang Tea	Green tea	6.30 ± 0.62	29.58 ± 2.4	2.86 ± 0.26	35.80 ± 3.26	13.13 ± 1.02	10.75 ± 0.96	30.47 ± 2.89	1.51 ± 0.08
Jieyang Chaoqing Tea	Green tea	4.86 ± 0.37	19.77 ± 1.29	7.05 ± 0.35	20.86 ± 1.86	16.40 ± 1.34	4.70 ± 0.37	34.02 ± 2.46	1.46 ± 0.09
Fenggang Zinc-Selenium-Enriched Tea	Green tea	5.18 ± 0.48	26.03 ± 2.1	5.64 ± 0.22	56.30 ± 4.59	14.04 ± 1.24	12.56 ± 0.92	47.78 ± 3.39	3.01 ± 0.19
Yihong Tea	Black tea	-	-	-	1.17 ± 0.08	0.77 ± 0.02	-	4.57 ± 0.22	-
Selenium-Enriched Black Tea	Black tea	-	-	-	1.37 ± 0.1	2.67 ± 0.12	-		-
Yingde Black Tea	Black tea	-	-	3.59 ± 0.24	1.02 ± 0.07	3.87 ± 0.23	-	13.03 ± 0.82	-
Wuyi Narcissus Tea	Oolong tea	9.84 ± 0.87	13.66 ± 1.12	4.11 ± 0.20	11.05 ± 1.07	3.39 ± 0.19	4.31 ± 00.39	7.12 ± 0.34	-
Fenghuang Danzong Tea	Oolong tea	3.72 ± 0.26	12.10 ± 1.01	-	41.24 ± 3.45	1.75 ± 0.1	6.26 ± 0.42	17.14 ± 0.94	2.44 ± 0.16
Black Fu Brick Tea	Dark tea	2.21 ± 0.12	6.56 ± 0.35	-	4.35 ± 0.34	2.58 ± 0.09	1.89 ± 0.1	3.18 ± 0.29	-
**Name**		**Category**	**Gallic Acid**	**Chlorogenic Acid**	**Caffeine**	**Ellagic Acid**	**Astragalin**	**Quercetin**	**Theaflavin**
Chaoqing Green Tea		Green tea	0.75 ± 0.06	-	27.25 ± 1.69	-	0.38 ± 0.02	-	-
Selenium-Enriched Chaoqing Green Tea		Green tea	1.26 ± 0.08	0.20 ± 0.01	24.82 ± 0.95	1.54 ± 0.14	0.72 ± 0.05	-	-
Selenium-Enriched Matcha		Green tea	0.90 ± 0.07	-	28.51 ± 2.19	-	0.41 ± 0.02	-	-
Matcha		Green tea	1.26 ± 0.11	-	26.16 ± 1.72	-	-	-	-
Fried Green Tea		Green tea	0.87 ± 0.07	0.14 ± 0.01	22.83 ± 0.93	-	1.71 ± 0.14	-	-
Liping Xiang Tea		Green tea	1.75 ± 0.1	0.17 ± 0.01	29.68 ± 1.45	2.08 ± 0.11	0.30 ± 0.01	-	-
Jieyang Chaoqing Tea		Green tea	1.44 ± 0.06	0.77 ± 0.03	29.46 ± 1.82	-	1.21 ± 0.09	-	-
Fenggang Zinc-Selenium-Enriched Tea		Green tea	2.27 ± 0.18	0.22 ± 0.02	35.99 ± 2.22	2.78 ± 0.17	2.11 ± 0.13	-	-
Yihong Tea		Black tea	4.22 ± 0.28	-	31.68 ± 2.17	1.92 ± 0.13	0.98 ± 0.07	-	0.34 ± 0.02
Selenium-Enriched Black Tea		Black tea	4.44 ± 0.21	-	29.42 ± 2.1	1.78 ± 0.16	1.39 ± 0.11	-	0.88 ± 0.07
Yingde Black Tea		Black tea	2.92 ± 0.15	0.12 ± 0.01	31.17 ± 1.79	-	-	0.63 ± 0.05	0.42 ± 0.02
Wuyi Narcissus Tea		Oolong tea	2.38 ± 0.13	-	21.18 ± 1.33	1.26 ± 0.09	0.53 ± 0.03	-	-
Fenghuang Danzong Tea		Oolong tea	4.02 ± 0.38	-	37.95 ± 2.84	1.50 ± 0.11	0.59 ± 0.04	-	-
Black Fu Brick Tea		Dark tea	3.15 ± 0.2	-	18.01 ± 0.92	-	0.55 ± 0.03	0.21 ± 0.01	-

DW, dry weight; -, means not detected.

**Table 4:** The in vitro antioxidant capacities and total phenolic contents of selected teas.

Name	Category	FRAP (µmol Fe^2+^/g DW)	TEAC (µmol Trolox/g DW)	TPC (mg GAE/g DW)
Chaoqing Green Tea	Green tea	1530.51 ± 22.40	1419.00 ± 3.14	132.07 ± 0.79
Selenium-enriched Chaoqing Green Tea	Green tea	2819.20 ± 22.63	1960.46 ± 23.69	173.46 ± 1.18
Selenium-Enriched Matcha	Green tea	2691.66 ± 12.93	1567.68 ± 18.83	160.96 ± 3.50
Matcha	Green tea	1315.20 ± 16.80	733.30 ± 19.09	110.82 ± 1.09
Fried Green Tea	Green tea	2736.91 ± 52.92	1421.22 ± 5.44	161.65 ± 0.39
Liping Xiang Tea	Green tea	3891.20 ± 75.60	1589.87 ± 23.23	219.99 ± 1.42
Jieyang Chaoqing Tea	Green tea	2535.77 ± 16.80	1432.31 ± 20.58	174.43 ± 0.79
Fenggang Zinc-Selenium-Enriched Tea	Green tea	3891.20 ± 64.65	2162.40 ± 24.51	251.65 ± 4.43
Yihong Tea	Black tea	1095.77± 36.99	849.18 ± 9.19	110.41 ± 0.07
Selenium-Enriched Black Tea	Black tea	1060.63 ± 17.10	872.48 ± 10.79	112.65 ± 0.73
Yingde Black Tea	Black tea	1377.77 ± 32.32	1003.96 ± 5.88	148.02 ± 1.50
Wuyi Narcissus Tea	Oolong tea	1460.91 ± 55.16	899.94 ± 14.12	114.56 ± 1.60
Fenghuang Danzong Tea	Oolong tea	2657.60 ± 40.41	1358.59 ± 9.54	165.20 ± 1.88
Black Fu Brick Tea	Dark tea	1248.34 ± 12.12	736.84 ± 3.11	90.05 ± 1.40

FRAP, ferric-reducing antioxidant power; TEAC, Trolox equivalent antioxidant capacity; TPC, total phenolic content.
